# Globular Shaped Polypyrrole Doped Well-Dispersed Functionalized Multiwall Carbon Nanotubes/Nafion Composite for Enzymatic Glucose Biosensor Application

**DOI:** 10.1038/s41598-017-16541-9

**Published:** 2017-11-23

**Authors:** Bishnu Kumar Shrestha, Rafiq Ahmad, Sita Shrestha, Chan Hee Park, Cheol Sang Kim

**Affiliations:** 1 Department of Bionanosystem Engineering, Chonbuk National University, 567 Baekjedaero, Deokjin-gu, Jeonju-si, Jeollabuk-do, 54896 Republic of Korea; 2 Division of Mechanical Design Engineering, Chonbuk National University, 567 Baekjedaero, Deokjin-gu, Jeonju-si, Jeollabuk-do, 54896 Republic of Korea; 3 School of Semiconductor and Chemical Engineering, Nanomaterials Processing Research Center, Chonbuk National University, 567 Baekjedaero, Deokjin-gu, Jeonju-si, Jeollabuk-do, 54896 Republic of Korea

## Abstract

Herein, we report preparation of a bio-nanohybrid material of homogenously dispersed functionalized multiwall carbon nanotubes (*f*MWCNTs) in Nafion (Nf) doped with polypyrrole (PPy) and followed by one-step *in situ* electrochemical polymerization along with glucose oxidase (GOx) on a platinum (Pt) electrode. The bioengineered Nf-GOx-*f*MWCNTs-PPy/Pt electrode showed excellent electrocatalytic performance to detect glucose with a high sensitivity (54.2 μAmM^−1^ cm^−2^) in linear range of up to 4.1 mM as well as a low detection limit of 5 μM (S/N = 3), response time within 4 s, good selectivity, stability, and practical applicability. It is our hope that the comprehensive results will contribute to design an efficient glucose biosensor with practical prospects for biomedical applications.

## Introduction

Over the past few decades, electrical stimulus-responsive organic polymers have been investigated, and their composite materials offer novel uses in energy storage, electrochromic displays, information memory, anti-static materials, anti-corrosives, fuel cells, electromechanical devices, biosensors, and biomedical devices^1–3^. Electroactive conducting polymers (ECPs) have been tailored to obtain superior electrical conductivity, thermal, and chemical stabilities at normal temperature and pressures. Also, ECPs can be synthesized as bioactive and biocompatible materials to employ them as a better alternative than metal and metal-oxide nanoparticles. ECPs are eco-friendly functional materials that can be used to fabricate biosensor devices. To date, various ECPs have been used to construct highly sensitive and selective glucose biosensors^4^. Among these, polypyrrole (PPy) has been extensively used to provide stable, facile, low-cost, biocompatible, and convenient materials for glucose biosensors^5^. Oxidized PPy has strong affinity to immobilize negatively-charged enzymes via its electrostatic attraction, and it has shown robust activity both with an electrocatalytic and remarkable sensitivity toward glucose using electrochemical techniques^6^. A large number of studies have been carried out to develop effective techniques for glucose biosensors application, which is important for early identification, monitoring, and comprehensive treatment of diseases including diabetes, kidney disease and thalassemia^7^. Taking this into account, biosensors based on electrochemical techniques promise reliability and accuracy in real time detection as well as high sensitivity and specificity to glucose^8^.

In recent years, the demand for electrochemical glucose biosensors has grown dramatically in biomedical, pharmaceutical, food industry, and biofuel cell applications due to their simplicity, cost effectiveness, widespread availability, and low power requirements and extreme precision^9^. Nano-engineered metal nanoparticles and their oxides integrated on PPy within carbon nanotubes (CNTs), carbon dots, and graphene oxide are also prominent materials that can be used to fabricate glucose biosensors^10–13^. The excessive use of metal oxide nanoparticles, while fabricating the sensing devices have many challenges, e.g. limited biocompatibility, time consuming for biofunctionalization, expensive, and also show adverse effects on ecological and biological toxicity. Furthermore, lack of dispersibility of pristine CNTs and their allotropes in aqueous or other organic solvents results in agglomeration and an increase in roughness of the biosensor electrode surface so that loading of biomolecules become less stable during the fabrication process^14,15^. These disadvantages and obstacles can successfully replace by metal-free and label-free nanohybrid composite materials, i.e. PPy-doped functionalized MWCNTs. The synthesis of high aspect ratio composite materials using ECPs through electrochemical polymerization for glucose biosensor application has been well studied^16,17^. The anodic polymerized PPy in the form of nanowires, nanoparticles and layered structures at a low applied voltage not only improves enzyme immobilization but also improves the sensing performance of enzymatic electrochemical-based glucose biosensors. Moreover, the inherent electroactive polymeric activities and a tendency to integrate with hydrogel-forming porous composite matrix ensure that PPy retains its benefits in biomedicine, engineering, and highly-sensitive amperometric immunosensors^17–19^.

Enzymatic glucose biosensors based on MWCNTs and its composite material has been introduced to glucose detection with electrochemical methods^20–22^. CNTs are ideal elements with a high surface area-to-volume ratio, and they play a pivotal role as biosensor electrode materials that enhance the electron transport rate, increase sensitivity, and electrocatalytic activity towards glucose oxidation during electrochemical analysis^23^. In addition, Cosnier *et. al*. described the π-stacking or non-covalent interactions between single-walled CNTs and biotin functionalized PPy composite to demonstrate the synergistic effects for excellent electrical conductivity and biocompatibility of the functional materials^24–26^. To enhance GOx amount that covalently immobilizes or entraps into intrinsically high aspect interfacial surfaces of nanosized one-dimensional CNTs can also be possible through their surface functionalization in acidic conditions. This attribute suggests that improvement of physicochemical stability of the composites could be improved. Moreover, PPy-decorated *f*MWCNTs containing carbonyl groups have a tendency to immobilize a high amount of enzymes using an electrochemical polymerization method^25,27^. It is noteworthy that a breakthrough began when *f*MWCNTs were uniformly dispersed in aqueous solution in the presence of Nf, resulting in a large active surface area for biosensor electrode fabrication^28,29^. The hydrophilic anionic polar group side chains in Nf have a high electron density, generating electrostatic repulsive forces and Ven Der Waals repulsion force between Nf and *f*MWCNTs, resulting homogenous dispersion of *f*MWCNTs. As a result, oxidized PPy can easily be doped on *f*MWCNTs with covalent bonding at a low applied voltage during polymerization. In addition, Nf exhibits better selectivity towards glucose in the presence of interfering species due to the electrostatic repulsion to the most negatively-charged electroactive species, and it also supports an increase in the enzyme loading capacity of functional composite films^30–32^. Therefore, it is highly desirable to develop stable, selective, and sensitive bio-nanohybrid composite-based enzymatic biosensor to overcome dip-coating and casting process methods.

**Figure 1:**
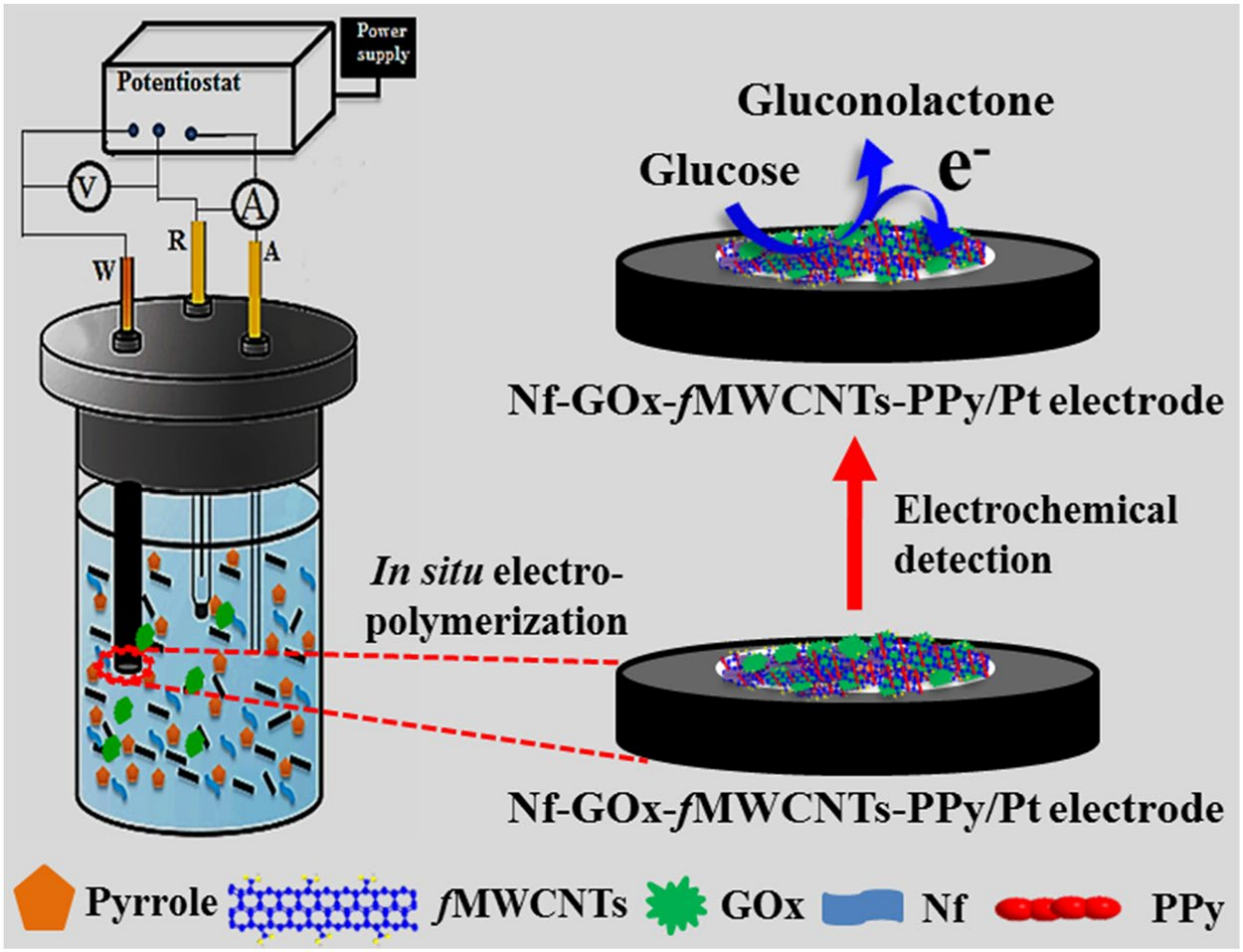
Schematic illustration of glucose biosensor fabrication via one-step *in situ* electrochemical polymerization method.

Here, we synthesized high conductance, large aspect ratio and electroactive bio-nanohybrid functional composite (Nf-GOx-*f*MWCNTs-PPy) materials. The aqueous suspension of the functional composite materials were deposited on a Pt working electrode using a simple one-step *in situ* electropolymerization technique to achieve a novel microarchitecture with precisely controlled large active surface area (Fig. 1; details are given in experimental section). The modified Pt electrode with new architecture has high electrocatalytic efficiency towards glucose oxidation and renown as the electrochemical based glucose biosensor. The regular shaped globular array of PPy doped on the surface of well-dispersed *f*MWCNTs in Nafion (Nf) provided a suitable avenue to immobilize or encapsulate large amount of GOx uniformly. Furthermore, Nf has potential ability to migrate protons in an aqueous phase, and this creates proton tunnelling within the sulfonate groups through narrow holes. As a result, the fabricated biosensor electrodes exhibited excellent performance in both ionic and electronic conductivity as well as high sensitivity during electrochemical detection of glucose. In addition, we were able to investigate the practical application of our purposed biosensor through addition of different glucose concentrations in human serum samples.

## Results and Discussions

### Morphological characterization of as-synthesized bio-nanohybrid composites

**Figure 2:**
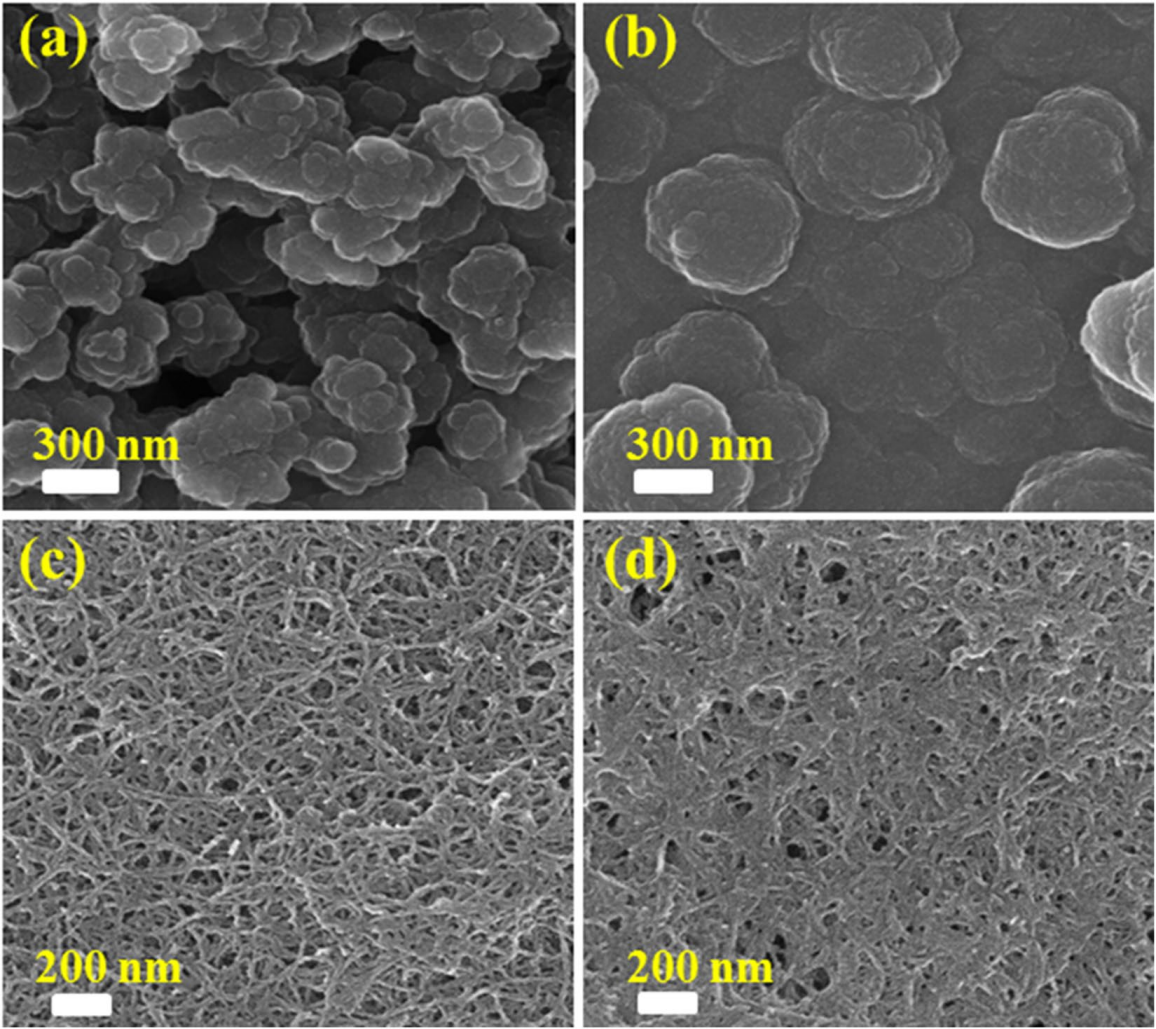
FE-SEM images of oxidized PPy (**a**), PPy-GOx (**b**), Nf-*f*MWCNTs-PPy (**c**), and Nf-GOx-*f*MWCNTs-PPy (**d**) after electropoymerization.

The morphological characteristics of nano-hybrid bio-composite films deposited on Pt disk electrode via electrochemical polymerization were characterized (Fig. 2). The FE-SEM image of oxidized PPy in Fig. 2a shows micro-globular, spherical and relatively high surface roughness. However, the GOx intercalation with the help of electrostatic forces occupies some porous space in the polymeric chain of the PPy film, which reduces a significant decrease in surface roughness, as shown in Fig. 2b. Well-dispersed *f*MWCNTs in Nf were decorated with globular shape PPy and modified in the form of spiral nanowires (Fig. 2c), where Nf plays a crucial role for the distribution of *f*MWCNTs. The uniformly-dispersed CNTs decorated with PPy create highly porous and large active surface area. After GOx encapsulation, the porosity of the film was occupied and displayed a homogenous and smooth surface of Nf-GOx-*f*MWCNTs-PPy composite film (Fig. 2d). We used electrochemical technique to determine the effective surface area of Pt electrode modified with Nf-GOx-*f*MWCNTs-PPy nanohybrid film (0.22 cm^2^), which was found to be much higher than effective surface area of unmodified Pt electrode (0.023 cm^2^). Detail of effective surface area measurements (Fig. S1) are given in the supplementary information.

**Figure 3:**
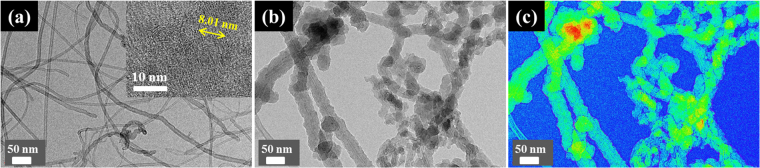
TEM images of functionalized MWCNTs (**a**), *f*MWCNTs-PPy (**b**), and mapping showing PPy doped *f*MWCNTs (**c**). Inset a shows HR-TEM image of *f*MWCNT.

Figure 3 shows the TEM images of *f*MWCNTs (a) and PPy-doped *f*MWCNTs (b and c). The inset in Fig. 3a shows HRTEM image of the *f*MWCNTs. The decrease in the external diameter of the nanotubes (~8.01 nm) is due to the destruction occurred on edge of nanotubes during the carboxylic group’s functionalization. The TEM image of the PPy-doped *f*MWCNTs clearly shows a uniform decoration of PPy (Fig. 3b). In addition, the elemental mapping image shows a green color around *f*MWCNTs surface, which indicates the electrodeposition of PPy array in Fig. 3c.

The typical XRD patterns of MWCNTs (curve a), *f*MWCNTs (curve b), PPy (curve c), and Nf-*f*MWCNTs-PPy composite matrix (curve d) are shown in Supplementary Fig. S2. The hexagonal graphite-like geometry of MWCNTs was assigned from the diffraction peaks at (002) and (101) planes, which corresponds to 2θ degree values at 25.59° and 44.34°, respectively (curve a). However, there was no distortion in crystal lattice of hexagonal graphite-like structure after acid functionalization of MWCNTs, which reveals that *f*MWCNTs retain their original geometry (curve b). Even though a less intense and small shift of peak from 43.84° to 42.21° in *f*MWCNTs indicate the successful functionalization of MWCNTs. A sharp peak appears at 27.6° (2θ) is ascribed to amorphous nature of oxidized PPy having inter-planar d spacing of 3.45 Å, resulting from the repeated unit of regular pyrrole rings that is oriented more regularly in a globular array (curve c)^33^. It is more interesting that a slight shift in the peak intensity towards a lower value of 2θ has been observed in the Nf-*f*MWCNTs-PPy composite (curve d), confirms that the *f*MWCNTs are well decorated with a PPy array which is in agreement with the HR-TEM image (Fig. 3b).

To elucidate the bonding configuration of PPy polymers integrated on *f*MWCNTs surface, FT-IR reflection spectra were investigated (Supplementary Fig. S3). In Fig. S3a, the spectrum of PPy showed a significant peak at 3171 cm^−1^ that can be attributed to N-H stretching and the broad bands around at 1400 cm^−1^ −1627 cm^−1^ correspond to C=C and C-C stretching^34^. The absorption peaks at 1347 cm^−1^ and 1207 cm^−1^ are assigned to C-C ring stretching and C-N deformation mode, respectively. Furthermore, peaks from 782 cm^−1^ to 848 cm^−1^ appeared due to C-C stretching and C-H out of plane deformation in heterocyclic aromatic rings of polymeric conjugation in PPy. As we can observe in Figure S3b, FT-IR spectrum of *f*MWCNTs shows strong bands intensity at 1740 cm^−1^ and 3595 cm^−1^ correspond to the C=O and O-H groups, respectively^35^. Figure S3c illustrates the conjugation of Nf-PPy-*f*MWCNTs composites during *in situ* polymerization, where π-electrons on sidewalls of CNTs have a tendency to form covalent bonds with bipolarons state of PPy which was obviously justified from the shifting of 2θ value in Fig. S2d. Furthermore, electron-rich *f*MWCNTs are prone to form electrostatic and polar bonds with a highly oxidized PPy. Importantly, absorption band of PPy at 1556 cm^−1^ shifts to 1578 cm^−1^ in the nanohybrid composite (Fig. S3c), confirming the perfect interfacial interaction (π-π conjugation) between *f*MWCNTs and PPy supported by Van Der Waals forces and enhances the charge transfer rate through Nf-*f*MWCNTs-PPy composite material^36^. Moreover, a small peak shift from 718 cm^−1^ to 823 cm^−1^ assigned to quinonoid bipolaronic structure of PPy and a peak at 3392 cm^−1^ is attributed to the formation of hydrogen bonds between the N-H stretching of PPy with carbonyl group of *f*MWCNTs that appeared in composite, indicating the formation of a nanohybrid film and resulting in the disappearance of C=O in *f*MWCNTs (Fig. S3c). A notable appearance of strong bands at around 1654 cm^−1^ (amide I) and 1532 cm^−1^ (amide II) confirms that GOx immobilized perfectly and reserves its activity within the bionanohybrid composite (Nf-GOx-*f*MWCNTs-PPy) after *in situ* electrochemical polymerization (Fig. S3d).

The UV-Vis absorption spectra depicted in Supplementary Fig. S4 were investigated to illustrate the incorporation of PPy on dispersed *f*MWCNTs. A noticeable peak appeared on *f*MWCNTs at 273 nm, corresponding to the successful functionalization of MWCNTs (Fig. S4, curve b). In addition, a higher peak intensity of *f*MWCNTs was observed as compared to pure MWCNTs (Fig. S4, curve a), which could be due to the presence of polar functional groups on CNTs surface walls. The band gap adsorption peaks at 230 nm and 320 nm (Fig. S4, curve c) ascribe to π-conjugation and π-π* transition in oxidized PPy originated from pyrrole rings, respectively^37^. The significance peak arises at 273 nm, confirming the presence of *f*MWCNTs on nanohybrid film (Fig. S4, curve d). However, similar peaks were highly dominant in Nf-*f*MWCNTs-PPy (Fig. S4, curve e), indicating that well-dispersed *f*MWCNTs are uniformly doped by PPy into nanohybrid composite in the presence of Nf. Moreover, higher band intensity of Nf-*f*MWCNTs-PPy exhibits excellent conductivity and is optically active due to PPy in bipolaron state that gives extra benefit for the fabrication of fluorescence biosensor.

### Electrochemical characterizations of surface modified Pt electrodes

**Figure 4:**
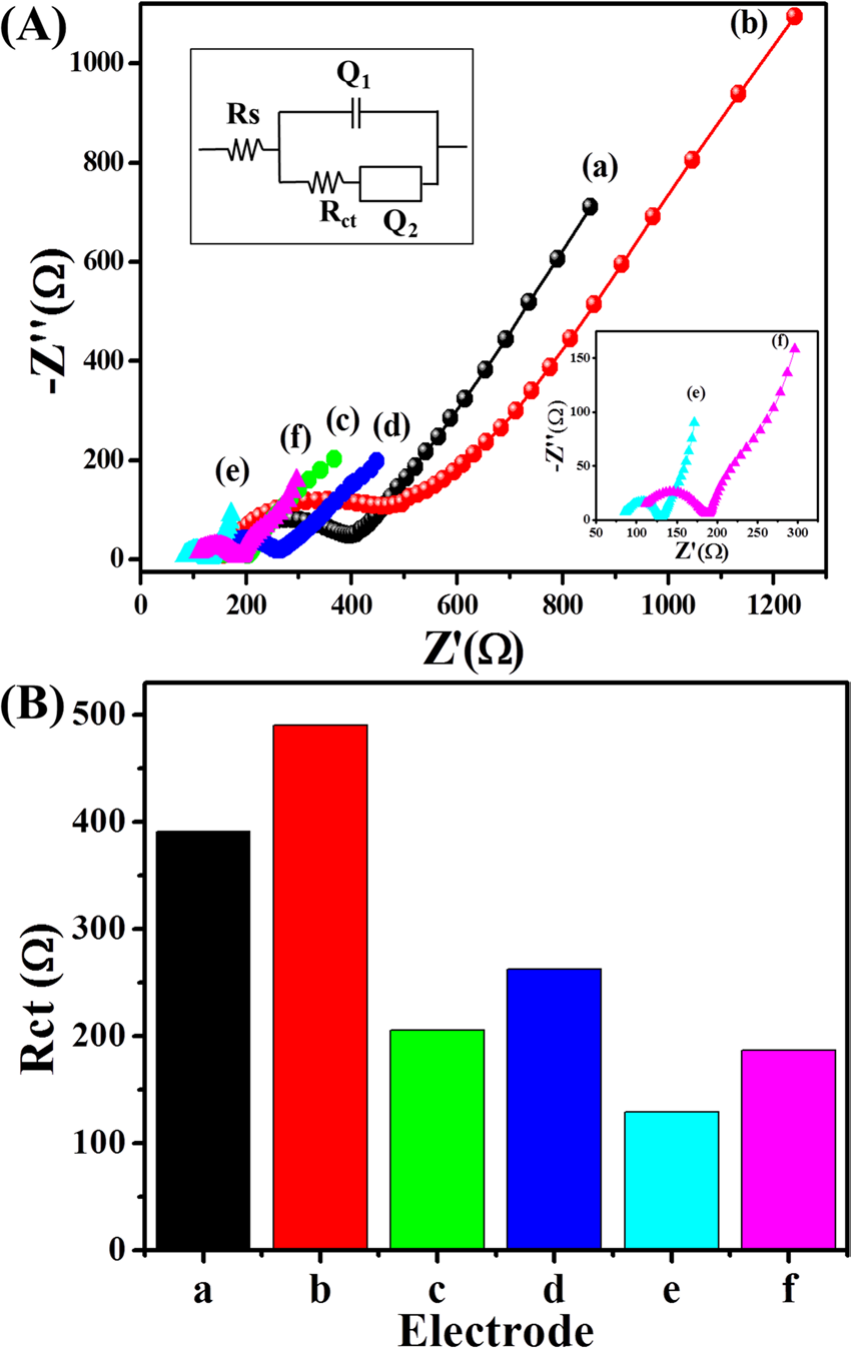
(**A**) EIS spectra obtained from Pt disk electrodes modified with PPy (a), GOx-PPy (b), *f*MWCNTs-PPy (c), GOx-*f*MWCNTs-PPy (d), Nf-*f*MWCNTs-PPy (e), and Nf-GOx-*f*MWCNTs-PPy (f) in 5.0 mM K_3_Fe[CN]_6_ containing 0.1 M KCl solution in 0.1 M PBS. (**B**) Histogram of R_ct_ values of different electrodes. In Fig. 4A, the upper inset shows the Randles equivalent circuit, and the lower inset shows the magnified view of the EIS spectra of Nf-*f*MWCNTs-PPy/Pt (sky blue color) and Nf-GOx-*f*MWCNTs-PPy/Pt (pink color).

To verify the excellent electron transport properties of the Nf-*f*MWCNTs-PPy nanohybrid composite film, we evaluated EIS obtained from of each modified electrode with different material as the frequency response with respect to electron interception and diffusion at electrode-electrolyte interface (Fig. 4). All impedance spectra consist of a semicircle portion in higher frequency region where the diameter quantifies charge-transfer resistance (R_ct_), which explains the charge transport kinetic at electrode-electrolyte interface and a linear sloping portion at lower frequencies related to the diffusion control process of the reactive species and conductivity of the materials. The obtained data was fitted using a Randles equivalent circuit, where Q_1_ represents the interfacial double layer capacitance, Q_2_ is a Warburg impedance, R_s_ is the bulk solution resistance, and R_ct_ is charge-transfer resistance of the materials (upper inset of Fig. 4A). From Fig. 4A, all spectra show small semi-circular diameters, indicating the superior electrical conductivity of the materials. Furthermore, the slope of the linear portion of all curves show the diffusion of the redox charged solution on an interfacial electrode surface due to the high aspect ratio. The R_ct_ values for different modified electrodes without GOx immobilization were calculated to be 390.7 Ω, 205.3 Ω, and 129 Ω, assigned to PPy (curve a), PPy-*f*MWCNTs (curve c), and Nf-fMWCNTs-PPy (curve e), respectively, and obtained R_ct_ values are also presented as histogram (Fig. 4B). The decrease in R_ct_ value of bio-nanohybrid film (Nf-*f*MWCNTs-PPy) is due to the synergetic effect of a superior conductivity of well-dispersed *f*MWCNTs that were uniformly doped with an oxidized PPy array in Nf, which makes a highly porous architecture in the film^38–40^. However, after GOx immobilization sequence of R_ct_ values for each modified electrodes are PPy-GOx (490.16 Ω, curve b) > GOx-*f*MWCNTs-PPy (262.6 Ω, curve d) >Nf-GOx-fMWCNTs-PPy (186.87 Ω, curve f). The increase in R_ct_ values and a decrease in slope of straight lines in the presence of GOx suggests strong encapsulation of enzymes, which act as a blocking agent for charge transport and electron transfer rate.

**Figure 5:**
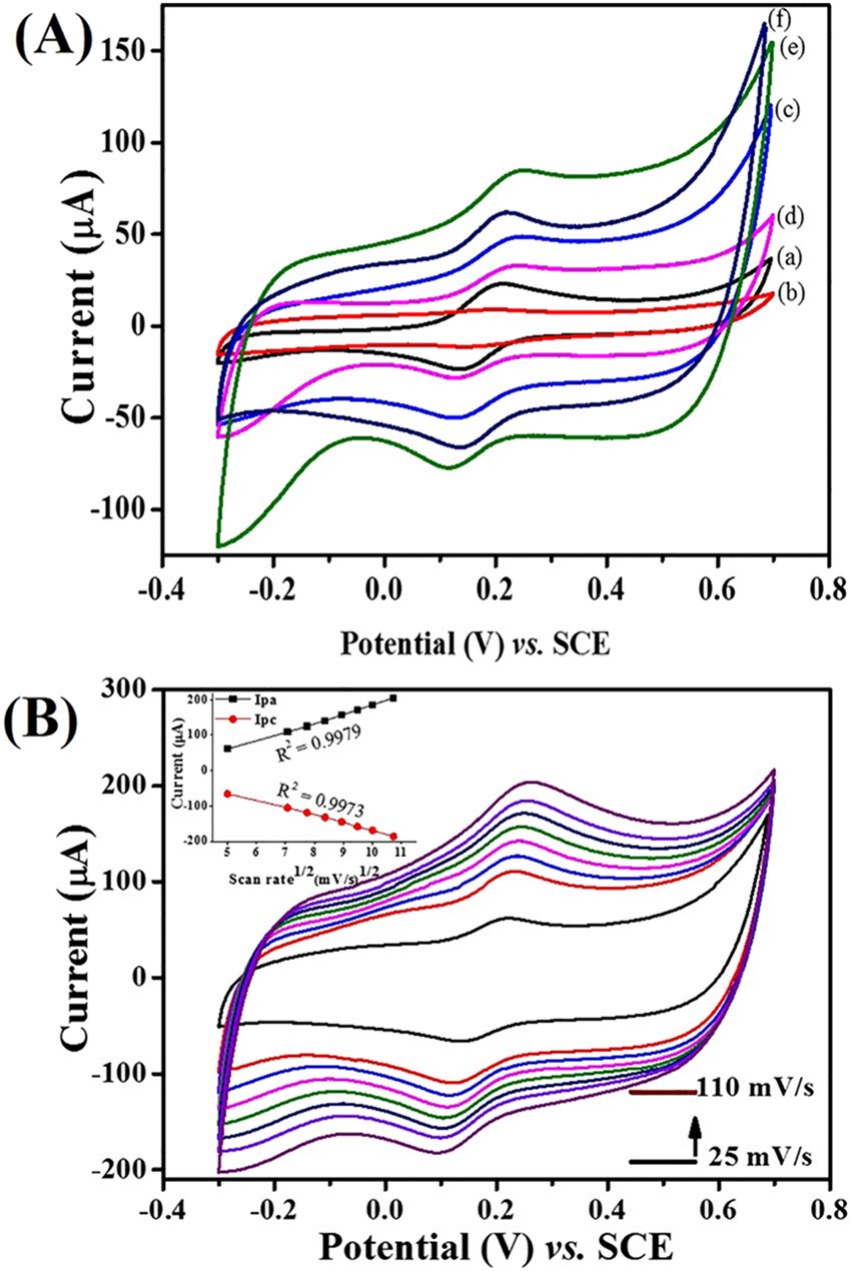
(**A**) CV response curves of different modified Pt electrodes, i.e., PPy/Pt (a), GOx-PPy/Pt (b), *f*MWCNTs-PPy/Pt (c), GOx-*f*MWCNTs-PPy/Pt (d), Nf-*f*MWCNTs-PPy/Pt (e), and Nf-GOx-*f*MWCNTs-PPy/Pt (f) in 5.0 mM K_3_Fe[CN]_6_ containing 0.1 M KCl in 0.1 M PBS. (**B**) CV curves of Nf-GOx-*f*MWCNTs-PPy/Pt electrode at different scan rates (25–110 mV/s) in 5.0 mM K_3_Fe[CN]_6_ containing 0.1 M KCl in 0.1 M PBS. Inset B shows the calibrated curve of Ipa and Ipc *vs*. square roots of scan rate.

To investigate the electrochemical behavior of each surface modified electrode, cyclic voltammetric (CV) responses were recorded in 5.0 mM K_3_Fe[CN]_6_ containing 0.1 M KCl prepared in 0.1 M PBS (pH 7.4) at a scan rate of 25 mV/s (Fig. 5A). Each modified electrode exhibited a pair of reversible redox peak currents that could be attributed to direct electron transfer, but quasi-reversible electrochemical behavior. The CV response of the Nf-*f*MWCNTs-PPy/Pt electrode shows a significantly higher redox peak current with an anodic peak potential (E_pa_) at 0.21 V and cathodic peak potential (E_ca_) at 0.14 V (curve e). However, Nf-*f*MWCNTs-PPy/Pt electrode showed a lower peak-to-peak potential separation (ΔEp = 67 mV) compared to *f*MWCNTs-PPy/Pt (~70 mV, curve c) and PPy/Pt (~74 mV, curve a) electrodes. The larger background current of Nf-*f*MWCNTs-PPy/Pt is due to uniform, highly dispersed CNTs that were well-decorated with PPy, which results in a larger electroactive and catalytic active surface area with sufficiently defective sidewalls of *f*MWCNTs. In addition, smooth film of Nf-*f*MWCNTs-PPy offers a high mechanical stability and a favorable microenvironment for GOx immobilization, which employs synergistic effects for glucose oxidation. Each modified electrode along with GOx shows decrease in redox peak currents (curves b, d, and f) indicating that a thin blocking layer of GOx was formed between electrode-electrolyte phases that hinders the diffusion process of ferricyanide ions on electrode surface. Furthermore, electrochemical behavior of Nf-GOx-*f*MWCNTs-PPy/Pt electrode was tested using 5.0 mM [Fe(CN)_6_]^3−/4−^ as a redox marker containing 0.1 M KCl prepared in 0.1 M PBS (pH 7.4) at different scan rates (25–110 mV/s), presented in Fig. 5B. The redox probe [Fe (CN)_6_]^3−/4−^ shows an average peak-to-peak separation (ΔE_pa_) of about 78 mV, suggesting a quasi-reversible electron transport process attributed to the presence of GOx. Moreover, both curves of anodic and cathodic peak currents *vs*. square root of scan rate (inset Fig. 5B) were linearly proportional with correlation coefficient (R^2^) values of 0.9979 and 0.9973 for anodic and cathodic peaks, respectively, suggesting a surface-confined electrochemical redox process by Nf-GOx-*f*MWCNTs-PPy/Pt electrode^41^.

**Figure 6:**
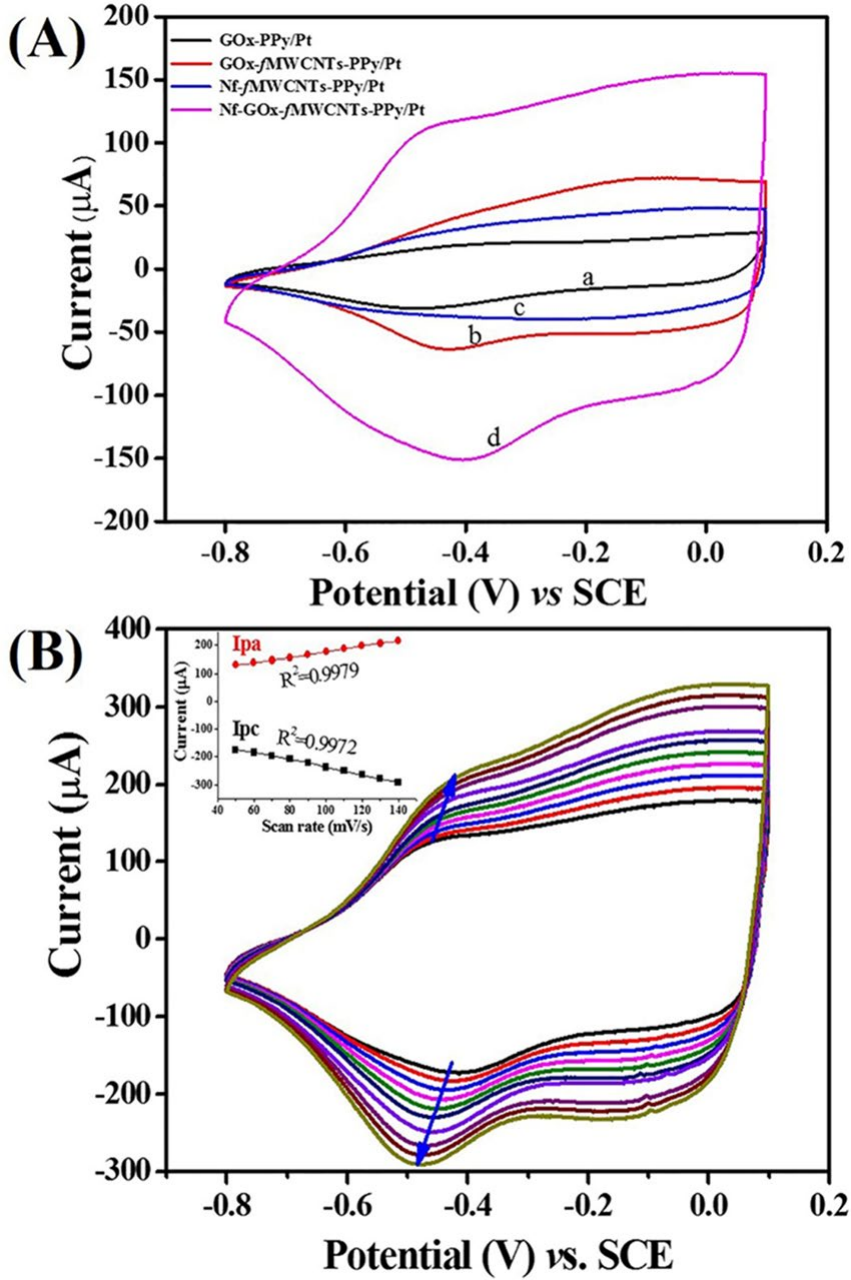
(**A**) CV response curves of GOx-PPy/Pt (a), (GOx-*f*MWCNTs-PPy/Pt (b), Nf-*f*MWCNTs-PPy/Pt (c), and Nf-GOx-*f*MWCNTs-PPy/Pt (d) in 0.1 M PBS at an applied potential range of −0.8 – + 0.1 V. (**B**) CVs of Nf-GOx-*f*MWCNTs-PPy/Pt electrode in 0.1 M PBS at different scan rate (50–140 mV/s). Inset B shows plot of redox peak currents *vs*. different scan rate.

Figure 6A illustrates the CV responses describing redox reaction behavior of GOx immobilized on electrodes, i.e. GOx-PPy/Pt, GOx-*f*MWCNTs-PPy/Pt, Nf-*f*MWCNTs-PPy (without GOx) and Nf-GOx-*f*MWCNTs-PPy/Pt in N_2_-saturated PBS (pH 7.4) solution. The redox peak current recorded for Nf-GOx-*f*MWCNTs-PPy/Pt electrode was higher than that for GOx-PPy/Pt and GOx-*f*MWCNTs-PPy/Pt electrodes. In addition, Nf-GOx-*f*MWCNTs-PPy/Pt electrode showed two well-defined redox peaks of reduction and oxidation at −0.45 V and −0.39 V, respectively. These average redox peaks were centered at −0.42 V and were taken as the surface formal potential (E°′) assigned to FAD-GOx/FADH_2_-GOx conversion, which is in consistent with the potential range reported in the literatures^42,43^. It is well known that a pair of redox peaks appeared in these potential ranges confirms the direct electron transfer from GOx, which illustrate the electrons transfer process between electrode surface and redox active center of FAD in GOx indicating quasi-reversible process^44–46^. Also, redox peak-to-peak separation of Nf-GOx-*f*MWCNTs-PPy/Pt electrode equal to 61 mV which is the characteristic of reversible electron transfer phenomenon on active center of GOx and surface-confined process. Importantly, a small value of redox peak-to-peak separation of Nf-GOx-*f*MWCNTs-PPy/Pt electrode as compared to GOx-*f*MWCNTs-PPy/Pt (97 mV) and GOx-PPy/Pt (121 mV) indicates a fast electron transfer process. The obtained results confirm that the architecture and bipolaron state of PPy forming Nf-*f*MWCNTs-PPy nanohybrid composite film has a tendency to immobilize large amounts of enzyme and has potential advantage of direct electron transfer capacity as compared to other electrodes, which makes it preferable to fabricate high-performance bio-electrocatalytic sensing devices^47^. The CVs of Nf-GOx-*f*MWCNTs-PPy/Pt electrodes at different scan rates (50–140 mV/s) were also recorded (Fig. 6B). The anodic (*I*_pa_) and cathodic (*I*_pc_) redox peak currents showed linear proportionality to the scan rates with a linear regression of 0.9979 and 0.9972 for *I*_pa_ and *I*_pc_ respectively (Fig. 6B, inset). Furthermore, the redox process of GOx in bio-nanohybrid composite film exhibited the reversible and surface-confined electrochemical process.

### CV measurements of glucose biosensor electrodes

**Figure 7:**
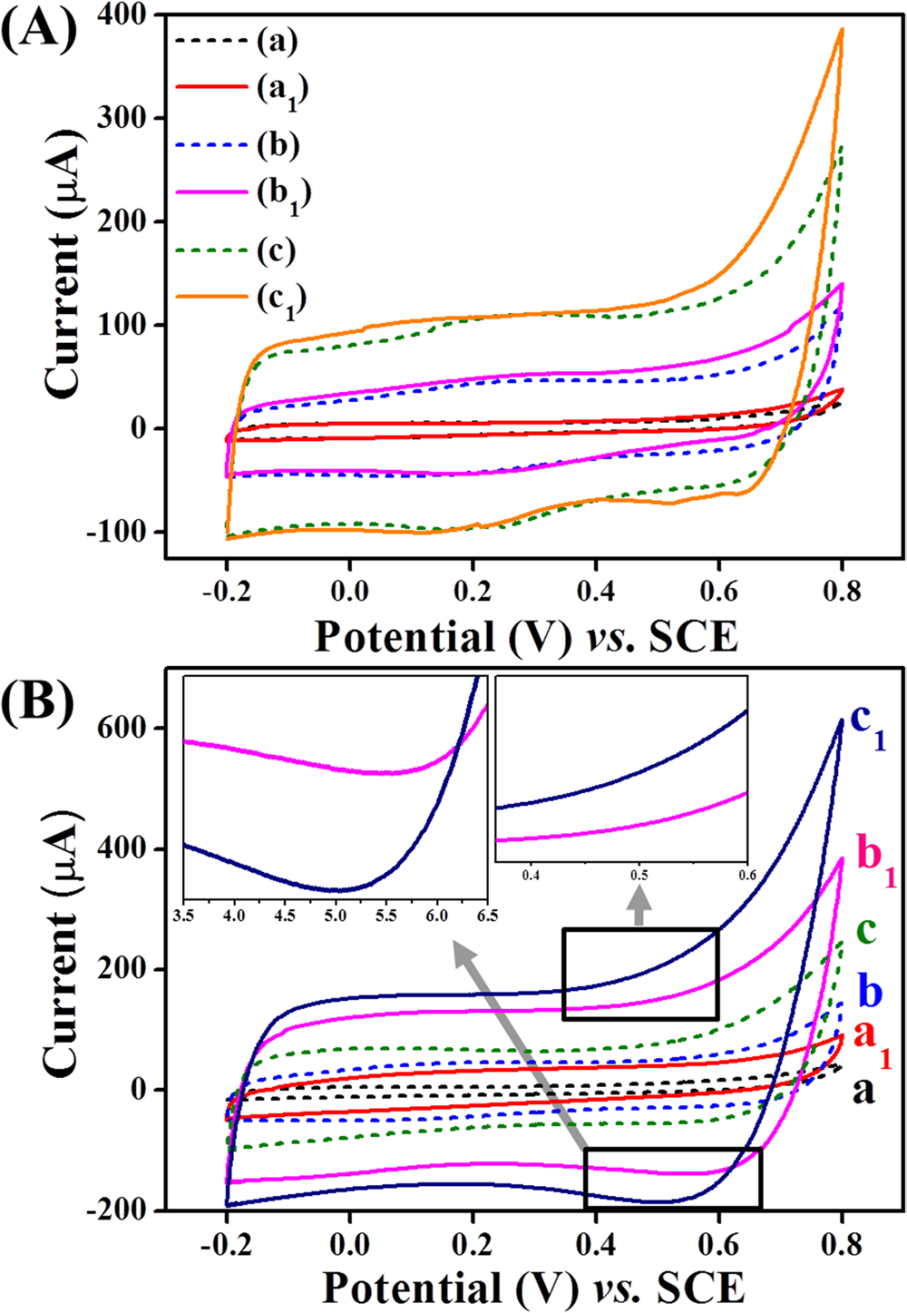
(**A**) CV response of PPy/Pt (curve a and a1), *f*MWCNTs-PPy/Pt (curve b and b1), and Nf-*f*MWCNTs-PPy/Pt (curve c and c1) electrodes measured in the presence of 0.05 mM glucose (solid lines) and in only 0.1 M PBS (pH 7.4) at 50 mV/s scan rate (short dash lines). (**B**) CVs of GOx immobilized electrodes i.e. GOx-PPy/Pt (curve a and a1), GOx-*f*MWCNTs-PPy/Pt (curves b and b1)), and Nf-GOx-*f*MWCNTs-PPy/Pt (curve c and c1) in the absence (short dash lines) and presence (solid lines) of 0.05 mM glucose in 0.1 M PBS (pH 7.4) at 50 mV/s scan rate.

The electrocatalytic performance of composites materials toward glucose detection were measured through CV technique using different electrodes, as shown in Fig. 7. The CVs obtained from different electrodes i.e. without GOx (Fig. 7A) and with (Fig. 7B) GOx immobilization were measured in 0.1 M PBS (pH 7.4) in the presence and absence of glucose. The CVs from different modified Pt-disk electrodes without GOx immobilization were expressed as solid lines (presence of 0.05 mM glucose) and short dash lines (absence of glucose) in Fig. 7A. From these response curves, there were no significant changes in *I*_pa_ and *I*_pc_ currents from the PPy film in the absence (Fig. 7A, curve a) and presence (Fig. 7A, curve a1) of 0.05 mM glucose. However, CVs obtained from the Nf-*f*MWCNTs-PPy/Pt electrode showed a higher background current (Fig. 7A, curve c1) compared to the *f*MWCNTs-PPy/Pt (Fig. 7A), curve b1) due to presence of Nf, where Nf has tendency to create a large active surface area of modified electrode after uniform dispersion of *f*MWCNTs. Even though, these nanohybrids could not exhibit a pronounced electrochemical and electrocaltalytic response for glucose oxidation.

Further, we recorded the CV responses from different electrodes after GOx immobilization on the composite materials in the presence and absence of glucose in 0.1 M PBS (pH 7.4), as shown in Fig. 7B. From the CV response curves, the redox current from GOx-PPy/Pt (Fig. 7B, curve a1) and GOx-*f*MWCNTs-PPy/Pt (Fig. 7B, curve b1) electrodes were smaller than current obtained from Nf-GOx-*f*MWCNTs-PPy/Pt (Fig. 7B, curve c1) electrode in the presence of 0.05 mM glucose. Interestingly, Nf provided a uniform and large effective surface area on modified electrode that enhances the capacity to integrate a higher amount of GOx. Also, the architecture of Nf-GOx-*f*MWCNTs-PPy nanohybrid material has abundant active sites due to the high aspect ratio of the *f*MWCNTs and well-dispersion of PPy decorated *f*MWCNTs forms an interfacial electronic configuration that employs the capacitive behaviour of the nanohybrid materials. The nanohybrid composite not only exhibits a higher electrical conductivity but it also has the ability to hold large capacitance currents. An enhanced capacitance current shows a dominant current response over the Faradic and redox currents during glucose oxidation. More importantly, we obtained a lower redox potential at 0.52 V (insets of Fig. 7B). The anodic current (264 µA) obtained during the oxidation of glucose by Nf-GOx-fMWCNTs-PPy/Pt electrode is ~3.17 times higher than current (80.8 µA) recorded in only 0.1 M PBS. The current increase can be attributed to the synergetic effect of electrical conductivity of completely-oxidized PPy-doped *f*MWCNTs in Nf and higher electrocatalytic function of large amount of GOx encapsulated into functional matrix. The possible mechanism for electrocatalytic oxidation of glucose can be expressed as follows^48^.

1 $${\rm{\beta }}\, \mbox{-} {\rm{D}} \mbox{-} {\rm{Glucose}}+{\rm{GOx}}( \mbox{-} {\rm{FAD}})\to {\rm{D}} \mbox{-} {\rm{Glucono}} \mbox{-} 1 \mbox{-} 5 \mbox{-} {\rm{lactone}}+\,{\rm{GOx}} \mbox{-} {{\rm{FADH}}}_{2}$$

2 $${\rm{GOx}} \mbox{-} {{\rm{FADH}}}_{2}+{{\rm{O}}}_{2}\to {\rm{GOx}} \mbox{-} {\rm{FAD}}+{{\rm{H}}}_{{\rm{2}}}{{\rm{O}}}_{{\rm{2}}}$$

3 $${{\rm{H}}}_{2}{{\rm{O}}}_{2}\to 2{\rm{H}}++{{\rm{O}}}_{2}+2{{\rm{e}}}^{-}$$

### Amperometric response of the fabricated biosensor

**Figure 8:**
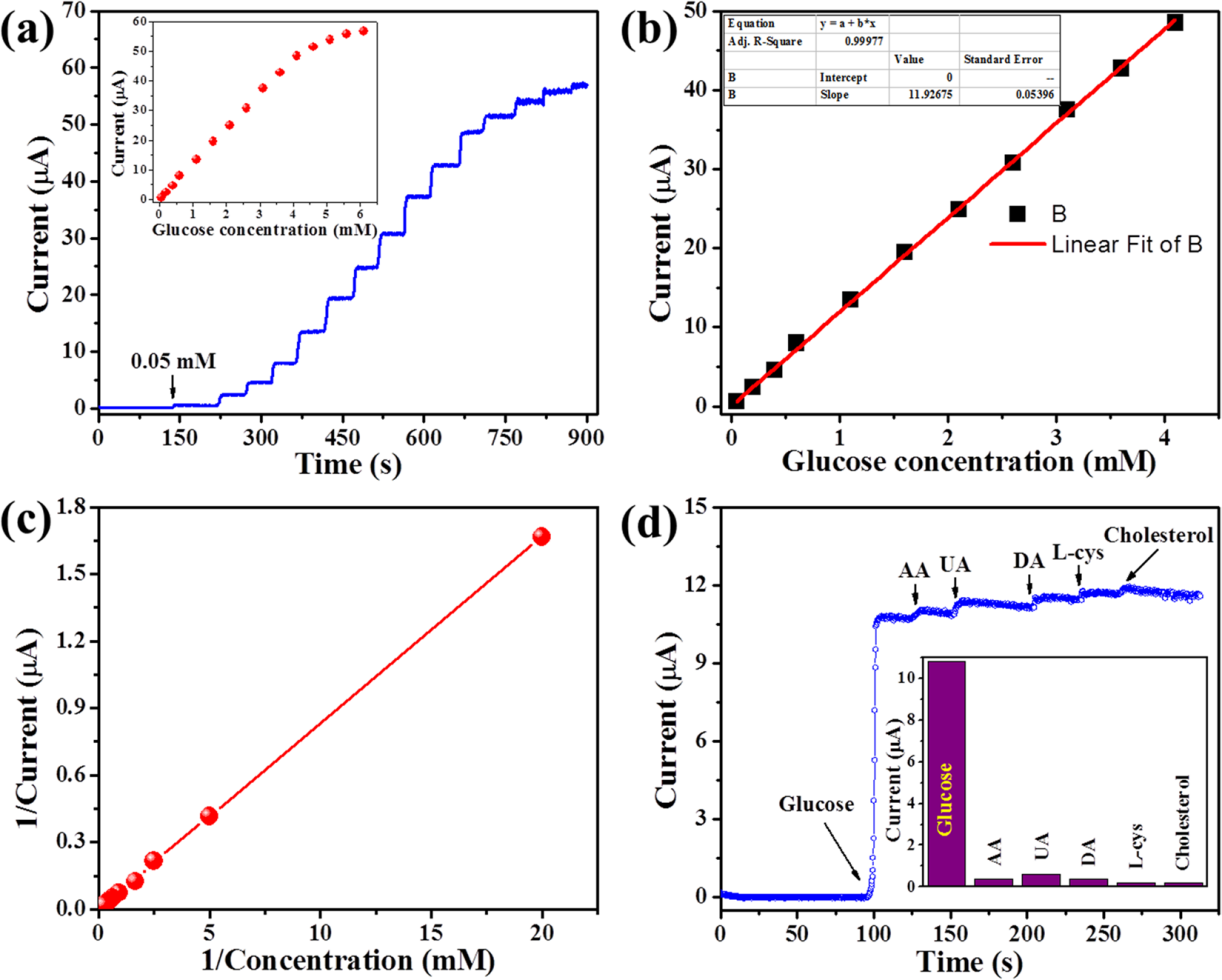
(**a**) Amperometric responses of the Nf-GOx-*f*MWCNTs-PPy/Pt electrode to successive addition of different concentration of glucose at constant applied potential of +0.52 *vs*. SCE in 0.1 M PBS (pH 7.4), (**b**) calibration plot i.e. current response *vs*. glucose concentration, (**c**) the Lineweaver-Burk plot, and (**d**) anti-inference test of biosensor with successive addition of 1 mM glucose and 0.1 mM of each interfering species i.e. AA, UA, DA, L-cys, and cholesterol. Inset (**a**) and (**d**) show the overall calibrated curve and histogram of anti-interference test, respectively.

To evaluate the sensing performance of the fabricated glucose biosensor, we measured the amperometric response of Nf-GOx-*f*MWCNTs-PPy/Pt electrode with a successive addition of different concentrations of glucose (0.05–6.6 mM) in 0.1 M PBS (pH 7.4) at an applied potential of +0.52 V *vs*. SCE under a stirring condition (Fig. 8). A rapid response was observed with every addition and as a result a stable step curve was obtained, which takes less than 4 s to reach 94% of the steady-state current (Fig. 8a). The corresponding calibration graph of the biosensor response shows a linear increase in the current response with an increase in the glucose concentration (inset, Fig. 8a). However, at a higher concentration a non-linear slope was obtained, indicating a saturation of the active site of the enzymes. In Fig. 8b, the linear plot (steady-state current *vs*. glucose concentration) obtained from Nf-GOx-*f*MWCNTs-PPy/Pt biosensor electrode showed a high sensitivity (54.2 µA cm^−2^ mM^−1^, calculated using effective surface area of modified electrode (0.22 cm^2^)) in the linear range of 0.05 to 4.1 mM with a high R^2^ of 0.9997). The lower limit of detection (LOD) was calculated to be 5.0 μM using a signal to noise ratio (S/N) of 3. Due to the excellent biocompatibility and electrocatalytic activity of Nf-GOx-*f*MWCNTs-PPy, the biosensor electrode holds high sensing performance compared to the previously-reported PPy based biosensors (Table 1). The apparent Michaelis-Menten constant (${{\rm{K}}}_{M}^{app}$) was calculated from the Lineweaver-Burk equation^49^:

$$1/{\rm{i}}={(K}_{M}^{app}{/i}_{\max })(1/C)+1/{{\rm{i}}}_{\max }),$$

where i, i_max_ and C correspond to the steady-state catalytic current after adding the substrate, maximum current measured under saturation of the substrate-enzyme reaction, and concentration of glucose, respectively. From the Lineweaver-Burk plot (Fig. 8c), the lower value of ${{\rm{K}}}_{M}^{app}$ (0.083 mM) was calculated, which is lower than mostly used ECPs based-biosensors^50,51^. The lower value of ${{\rm{K}}}_{M}^{app}$ ensures a high affinity of the enzyme to the substrate, which is essential for enzymatic-based biosensors and confirm the homogenous integration of the enzyme on the nanohybrid film.

**Table 1:** Comparison of our biosensor performance with other PPy-based biosensors.

Working electrode	Sensitivity (µAcm^−2^ mM^−1^)	${{\bf{K}}}_{{\boldsymbol{M}}}^{{\boldsymbol{a}}{\boldsymbol{p}}{\boldsymbol{p}}}$ (mM)	LOD (μM)	Linear range (mM)	Ref.
GOD/PPY-HRP-FCA/CCE	0.33	0.026	10	0.08–1.3	58
Al_2_O_3_/Pt/PPy/GOx	7.4	7.01	10	0.5–10	59
GOx/Pt-PAMAM-PPy	164	—	10	0.002–0.6	60
PPY/GOx/SWCNTs-PhSO_3_^−1^/PB	6	—	10	0.02–6	61
Pt/PPy/GOx	0.007	37.6	—	0–10	62
Nf-GOx-*f*MWCNTs-PPy/Pt	54.2	0.083	5	0.05–4.1	This work

### Anti-interference test, reproducibility and stability study

The selectivity of the fabricated Nf-GOx-*f*MWCNTs-PPy/Pt glucose biosensor electrode was tested in the presence of common interfering species such as ascorbic acid (AA), uric acid (UA), dopamine (DA), L-cysteine (L-cys), and cholesterol. The amperometric response of the biosensor was measured in 0.1 M PBS (pH 7.4) after addition of 1.0 mM glucose and 0.1 mM of each interfering species. As shown in Fig. 8d, addition of 1.0 mM glucose showed a rapid response. However, 0.1 mM addition of each interfering species showed a negligible response compared to that of glucose (inset, Fig. 8d). Moreover, we checked the effect of 0.01 mM of DA on CVs responses along with 0.05 mM glucose but no significant change occurred on the current response as compared to CV response from only glucose prepared in PBS (Supplementary Fig. S5). Thus, a good selectivity may enlist this glucose biosensor electrode to exhibit a high reliability in detecting glucose in blood serum samples.

The reproducibility and repeatability of the biosensor were examined by measuring the response current from six similarly fabricated biosensor electrodes. The relative standard deviation (RSD) obtained from an amperometric determination of 1.0 mM glucose was 3.2% at an applied potential of +0.52 V. Furthermore, stability of biosensor electrodes was investigated by the amperometric method after storing at 4 °C in 0.1 M PBS (pH 7.4) and measuring the current response every 3 days. The biosensor electrode showed almost no change for 15 days, and it retains ~96% of its original response over a storage period of 45 days (Supplementary Fig. S6).

### Real sample analysis

The applicability of the proposed glucose biosensor (Nf-GOx-*f*MWCNTs-PPy/Pt) electrode was investigated. The glucose concentration was determined in human serum samples after diluting it in different concentrations in 0.1 M PBS (pH 7.4). Then, the recovery of glucose was measured using standard addition of a known concentration of pure glucose in the serum samples (Table 2). From Table, the obtained glucose concentrations were in good agreement with known concentrations of glucose (4.89 mM) in human serum (Sigma-Aldrich, H4522)^52^. Therefore, the proposed biosensor showed excellent reliability and accuracy for glucose detection in a real sample. Additionally, we checked long term stability of our sensor electrode in the serum (Supplementary Fig. S7). For long term stability measurement using amperometric method, we took 9.5 mL PBS in electrochemical cell and added 0.5 mL serum. The amperometric response of Nf-GOx-*f*MWCNTs-PPy/Pt electrode remains unchanged over a continuous 10 min period, indicating long term stability of our electrode for glucose detection in serum.

**Table 2:** Glucose detection in real serum samples.

Samples	Concentration (mM)	RSD (%) (n = 3)	Added glucose (mM)	Recovery (%)
1	0.61	2.6	1.0	101
2	1.22	1.3	1.0	97
3	2.45	3.2	1.0	99

## Conclusions

In summary, a bionanohybrid material with controllable morphology was successfully synthesized via *in situ* electrochemical polymerization on the Pt electrode to obtain Nf-GOx-*f*MWCNTs-PPy/Pt modified electrodes and used as a glucose biosensor electrode. Nf in the composite material was used to facilitate the uniform dispersion of *f*MWCNTs. Also, the oxidized PPy grown in the defect sites of the *f*MWCNTs ensures a large number of active sites of *f*MWCNTs and PPy, which provides sufficient space for GOx immobilization. The optimal thickness of Nf-GOx-*f*MWCNTs-PPy serves as a novel, highly efficient and durable bio-functional electrocalalytic active material for glucose oxidation. Moreover, the Nf prevents GOx leaching and improves the physicochemical stability and preserves the bioactivity under the long-term storage of the biosensor electrode. In addition, the bioengineered electrode exhibits a spatially-biocompatible environment and excellent electrocatalytic activity to enable the direct electron transfer from GOx to the electrode surface. The fabricated biosensor electrode showed excellent performance, including a high sensitivity (54.2 μAmM^−1^cm^−2^) in a linear range of up to 4.1 mM, LOD of ~5.0 μM, fast response time (within 4s), good selectivity, excellent stability, and reproducibility for glucose detection. On the basic of experimental results and analysis, our proposed biosensor showed good reliability for glucose detection in a real serum sample. Thus, suggesting a promising applicability for glucose monitoring in real samples, which would pave the way for impressive performance in a routine analysis.

## Methods

### Materials

Pyrrole above 99% purity was obtained from Daejung-Korea. MWCNTs (Ca. ~10 nm in external diameter) synthesized via chemical vapor deposition (CVD) were purchased from Nanosolutions Co. Ltd., Korea. Glucose oxidase (GOx, EC 1.1.3.4, Type X-S 127 unit/mg) lyophilized powder, from Aspergillus niger, human blood serum (H4522), Nafion (Nf, 5 wt. % in lower aliphatic alcohol), L-cysteine (L-cys), and cholesterol were purchased from Sigma-Aldrich, Korea. β-D-Glucose and ascorbic acid (AA) were purchased from Tokyo Chemical Industry Co., Ltd. Dopamine (DA) and uric acid (UA) were obtained from Bioshop Canada Inc. Disodium hydrogen phosphate (Na_2_HPO_4_), monobasic potassium phosphate (KH_2_PO_4_), sodium chloride (NaCl), potassium chloride (KCl), sulphuric acid (H_2_SO_4_), nitric acid (HNO_3_), and acetonitrile (CH_3_CN) were obtained from Samchun Pure Co. Ltd., Korea. Phosphate buffer solution (PBS, 0.1 M, pH 7.4) was prepared in ultra-pure water purified by Millipore-Q system (18 MΩ cm). All chemicals and reagents were of analytical grade and were used as received without further purification.

### Fabrication of bio-nanohybrid composite based glucose biosensor

To fabricate the glucose biosensor electrodes, bare Pt electrodes having geometric area of 0.02 cm^2^ were consecutively polished with alumina slurries (0.3 µm and 0.05 µm), followed by diamond suspensions (0.25 µm) on a Rayon polishing pad. All polishing steps required extensive rinsing before treatment with sonication in ethanol for 15 min. The electrodes were washed and treated using cyclic voltammetry (CV) in an applied potential range of −0.2 to 1.0 V (*vs*. SCE) till constant CV curves were obtained in 0.5 M H_2_SO_4_ electrolytes, and the electrodes were dried under nitrogen (N_2_) atmosphere. Before making the bio-nanohybrid composite, pure MWCNTs were treated to generate more carbonyl and hydroxyl groups on the surface walls of CNTs. 0.5 g pristine MWCNTs were dispersed with a 3:1 wt % mixture of conc. H_2_SO_4_ (90 mL) and conc. HNO_3_ (30 mL) for 15 min via sonication. Then, the solution was transferred into a reflux condenser and was heated at 70 °C for 12 h to complete the surface functionalization^53^. After completing reaction, the mixture was allowed to cool down at room temperature, followed by filtration and continuous washing with double-distilled water to get *f*MWCNTs as a residue having a pH of 7.4.

In the next step, 0.05 M pyrrole in aqueous acetonitrile (1 M) solution containing 0.5 mg/mL GOx, 1.0 mg/mL *f*MWCNTs, and 50 μL of 0.5% Nf were electrochemically polymerized on the Pt electrode in a single-step using a three-electrode electrochemical system (CV technique), as shown in schematic (Fig. 1). The bio-nanohybrid (Nf-GOx-*f*MWCNTs-PPy) composite was obtained after 15 cycles (an optimized number of cycles) of CV scans in a fixed potential range from −0.15 to +0.8 V (*vs*. SCE) at a 25 mV/s scan rate, as shown in Supplementary Fig. S8a. The thickness of the polymeric composite is proportional to the number scan cycles. The increase in scan number may cause longer inter-facial diffusion distance between biomarkers and transducer. As a result transfer of electrons produce from the electrocatalytic reaction through electrode was hindered and the redox current response decreased. In contrast, the lower number of scan cycles were not able to entrap/attach sufficient polymeric nanomaterial/enzyme. Also, the enzyme may easily percolate or leach through the porous film during electrochemical measurements and may cause loss in catalytic behaviour of biosensor. Thus, we have optimized the scan number to obtain the maximum oxidizing current using CV technique in the presence of 0.05 mM glucose (Supplementary Fig. S8b). The facile polymerization encapsulates GOx within the PPy decorated Nf-*f*MWCNTs film during the anodic oxidation of pyrrole^54–57^. The fabricated Nf-GOx-*f*MWCNTs-PPy/Pt electrodes were rinsed with distilled water to remove loosely attached GOx, PPy, pyrrole and other materials. The biosensor electrodes were stored at 4 °C in 0.1 M PBS (pH 7.4) for further utilization. Other electrodes modified with different composite materials were also prepared using the same process.

### Physicochemical characterizations

The morphological properties of the as-prepared functional materials were characterized using field-emission scanning electron microscopy (FE-SEM, Carl Zeiss SUPRA 40VP, Germany) and transmission electron microscopy (HR-TEM, JEOL 2010, Japan). X-ray diffraction (XRD) patterns were obtained using an X-ray diffractometer (Rigaku, Japan) with high-intensity monochromatic Cu-Kα radiation as an incident beam (λ = 1.54 Å) over a Bragg’s angle range from 10° to 90°. The formation of nanohybrid composites and a bonding configuration for each of the composite samples was recorded using Fourier transform infrared spectrometry (FT-IR Perkin Elmer, Spectrum GX, USA). The UV-vis absorption spectra were measured using a UV/Vis//NIR spectrophotometer (Jasco V-670, Japan). Each electrochemical analysis was performed on an electrochemical workstation ZIVE SP1 Potentiostat/Galvanostat/EIS from WonATech Co. Ltd. Seoul, Korea. The conventional three-electrode configuration was adjusted using platinum (Pt) as a working bio-electrode modified with different nanohybrid materials, the Pt wire was used as a counter electrode, and saturated calomel electrode (SCE) was used as reference electrode. The potentiostatic electrochemical impedance spectroscopy (EIS) was recorded from each bionanohybrid composite based biosensor electrode using 5.0 mM K_3_Fe[CN]_6_ as redox probe containing 0.1 M KCl prepared in 0.1 M PBS (pH 7.4) at an amplitude of 10 mV and zero bias potential in a frequency range from 1 MHz to 1 Hz. All analytical solutions were purged with high-purity N_2_ for 30 min prior to each measurement.

## Electronic supplementary material

Supplementary information
