# Supplementary material for: Globular Shaped Polypyrrole Doped Well-Dispersed Functionalized Multiwall Carbon Nanotubes/Nafion Composite for Enzymatic Glucose Biosensor Application
Source: Sci Rep. 2017 Nov 23;7:16191. doi: 10.1038/s41598-017-16541-9 (PMC5701076; doi:10.1038/s41598-017-16541-9)
Supplement: Supplementary file 1 — Supplementary information [file 41598_2017_16541_MOESM1_ESM.pdf]

Supplementary information

Globular Shaped Polypyrrole Doped Well-Dispersed Functionalized Multiwall Carbon Nanotubes/Nafion Composite for Enzymatic Glucose Biosensor Application

Bishnu Kumar Shrestha[1,2], Rafiq Ahmad[3], Sita Shrestha[1], Chan Hee Park[1,2,*], Cheol Sang Kim[1,2,*]

[1]Department of Bionanosystem Engineering, Chonbuk National University, 567 Baekjedaero, Deokjin-gu, Jeonju-si, Jeollabuk-do, 54896, Republic of Korea.

[2]Division of Mechanical Design Engineering, Chonbuk National University, 567 Baekjedaero, Deokjin-gu, Jeonju-si, Jeollabuk-do, 54896, Republic of Korea.

[3]School of Semiconductor and Chemical Engineering, Nanomaterials Processing Research Center, Chonbuk National University, 567 Baekjedaero, Deokjin-gu, Jeonju-si, Jeollabuk-do, 54896, Republic of Korea.

---

* Authors to whom correspondence should be addressed.

E-mail: chskim@jbnu.ac.kr, biochan@jbnu.ac.kr, Tel: - +82-63-270-4284, Fax:-+82-63-270-2460

1. Determination of effective surface area of the modified electrode

To evaluate the effective surface area of the modified electrode, we used electrochemical technique. The electroactive effective surface area of Pt modified electrode with Nf-GOx-*f*MWCNTs-PPy or without (bare Pt electrode) functional material were calculated from *I*pa current obtained from cyclic voltammograms (CVs) recorded in aqueous solution of 5.0 mM $K_3Fe[CN]_6$ containing 0.1 M KCl with 0.1 PBS at different scan rate. The Peak currents (*I*pa) of both electrodes, as shown in Fig. S1a and S1b were proportional to the square root of scan rate. The *I*pa current from CV was used to determine the effective surface area of the electrode using Randles-Sevcik equation in redox process [1, 2]:-

$$Ipa = 2.26 \times 10^5 \times n^{3/2} \times A_{eff} \times \sqrt{D} \times \sqrt{V} \times C_0 \qquad (I)$$

where D, A_{eff}, n, V, and C_0 refer to diffusion coefficient (7.26×10^{-6} cm^2 s^{-1}) of 5.0 mM $K_3Fe[CN]_6$ in 0.1 M KCl, effective surface area of the electrode, number of electron (n=1) involves in half reaction, scan rate at which the potential swept and the concentration of the $K_3Fe[CN]_6$ in solution, respectively[3]. The linear plot of *I*pa vs. $\sqrt{V}$ was applied to obtain slope 7.98×10^{-4} AV$^{-1/2}$ S$^{1/2}$ for modified electrode and 8.5×10^{-5} AV$^{-1/2}$ s$^{1/2}$ for bare Pt electrode[4]. These slop values were used to calculate the effective surface area of modified electrode (0.22 cm^2) and unmodified electrode (0.023 cm^2) using above relation (I), where the electroactive surface area of modified electrode was ~9.5 times larger than unmodified Pt electrode surface area. Thus, the higher surface area of Pt modified electrode with Nf-GOx-*f*MWCNTs-PPy has good performance with better sensitivity of biosensor towards glucose during electrochemical detection.

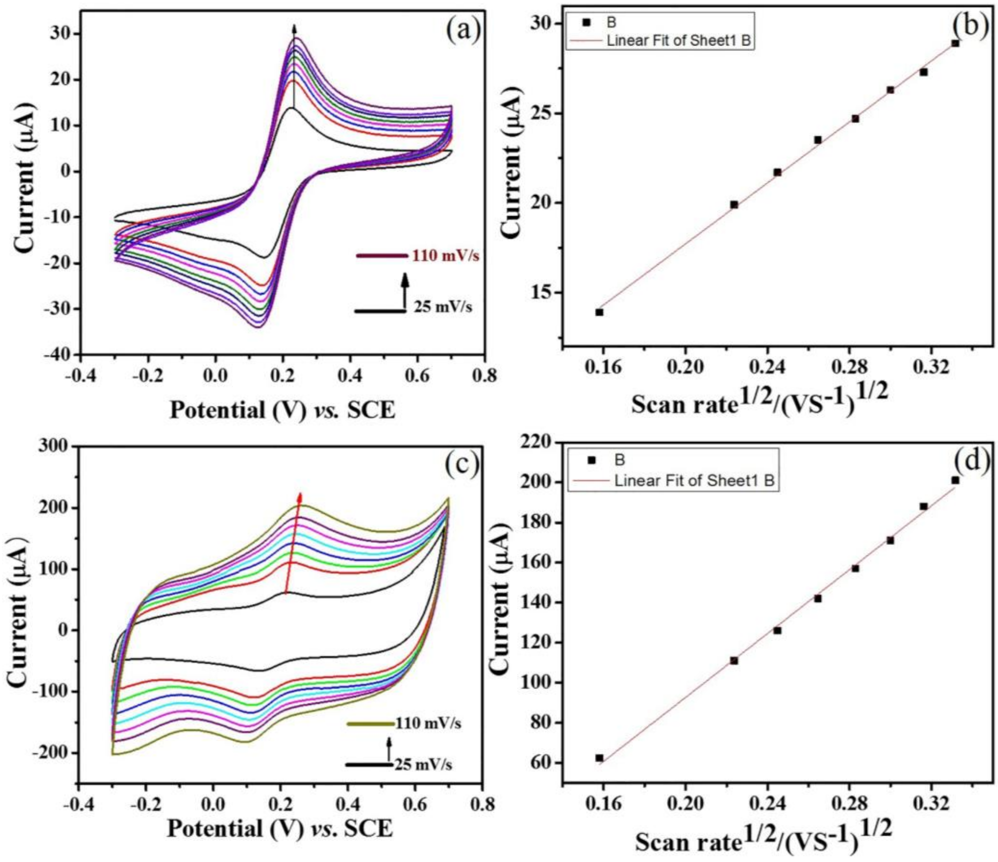

Supplementary Figure S1. CVs obtained from bare Pt (a) and Nf-GOx-*f*MWCNTs-PPy modified Pt electrode (c) in 5.0 mM $K_3Fe[CN]_6$ containing 0.1 M KCl prepared in 0.1 M PBS at different scan rates (25-110 mV/s). Plots (b) and (d) correspond to linear relation curves between *I*pa *vs.* square root of scan rate (V/s) for unmodified Pt electrode and modified Pt electrode, respectively.

2. XRD, FT-IR, and UV-Vis measurements

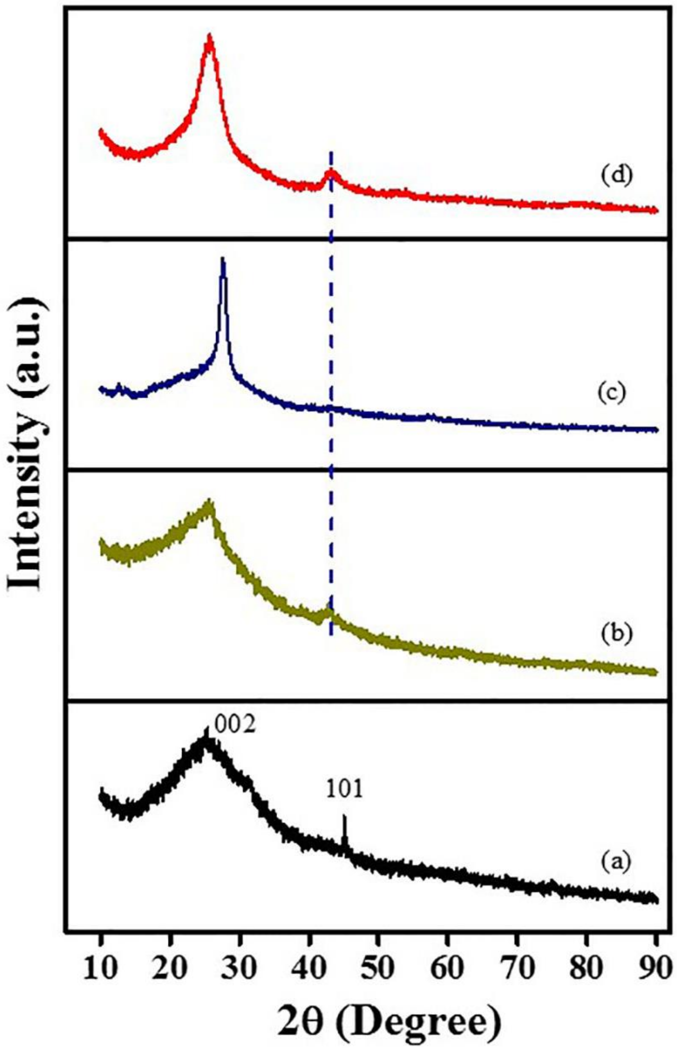

Supplementary Figure S2. XRD spectra of pristine MWCNTs (a), *f*MWCNTs (b), PPy (c), and Nf-*f*MWCNTs-PPy (d).

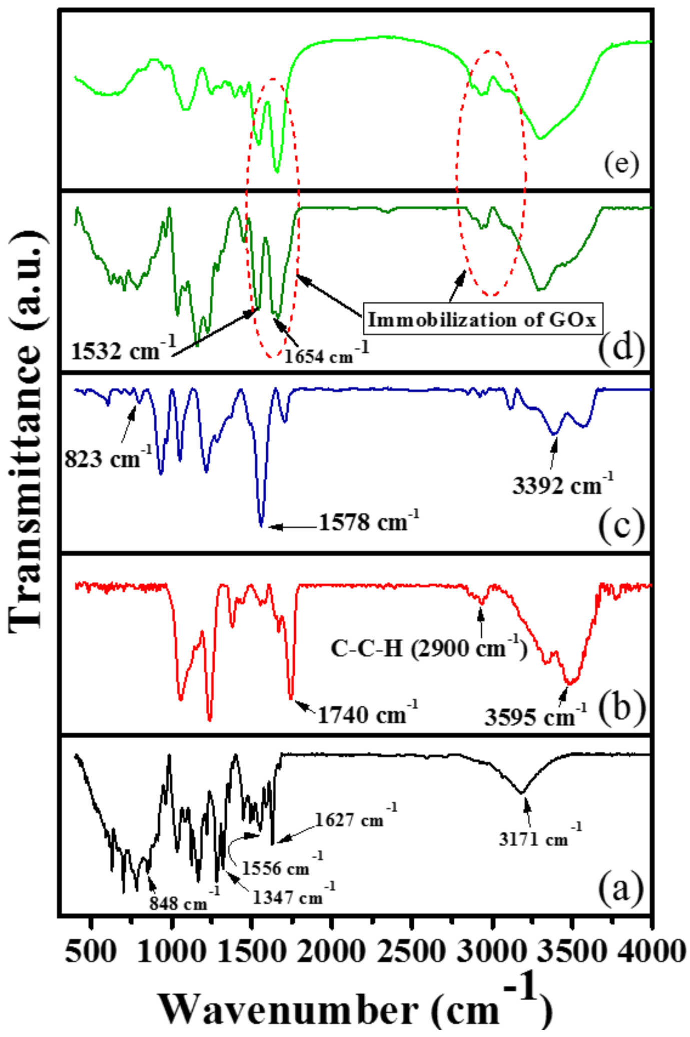

Supplementary Figure S3. FT-IR spectra of electropolymerized PPy (a), *f*MWCNTs (b), Nf-*f*MWCNTs-PPy (c), Nf-GOx-*f*MWCNTs-PPy (d), and pure GOx (e).

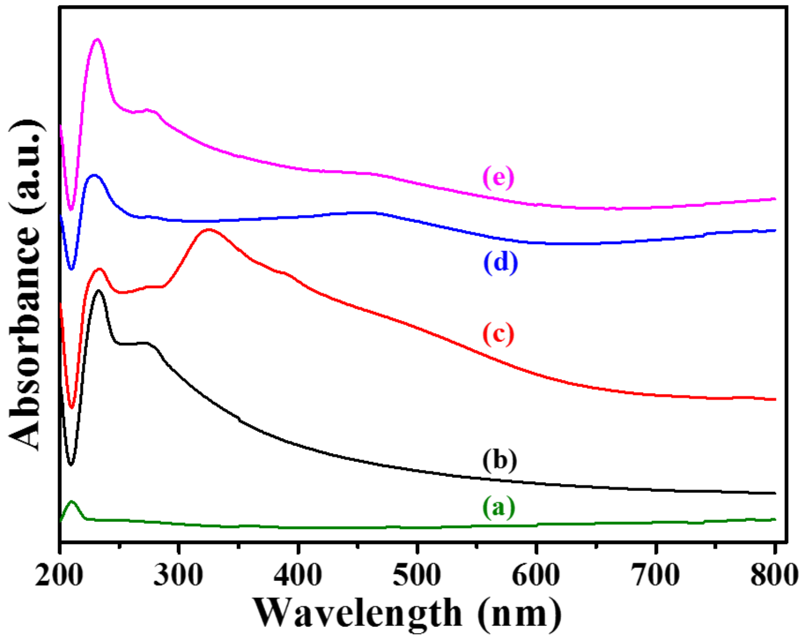

Supplementary Figure S4. UV-Vis spectra of pristine MWCNTs (a), *f*MWCNTs (b), PPy (c), *f*MWCNTs-PPy (d), Nf-*f*MWCNTs-PPy (e).

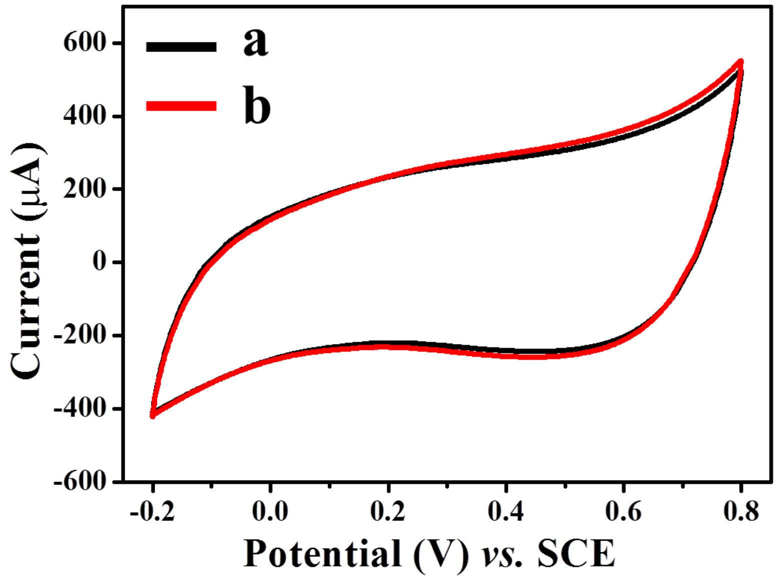

Supplementary Figure S5. CV response curves in 0.1 M PBS (pH 7.4) containing 0.05 mM glucose only (curve a, black line) and with 0.01 mM dopamine (curve b, red line).

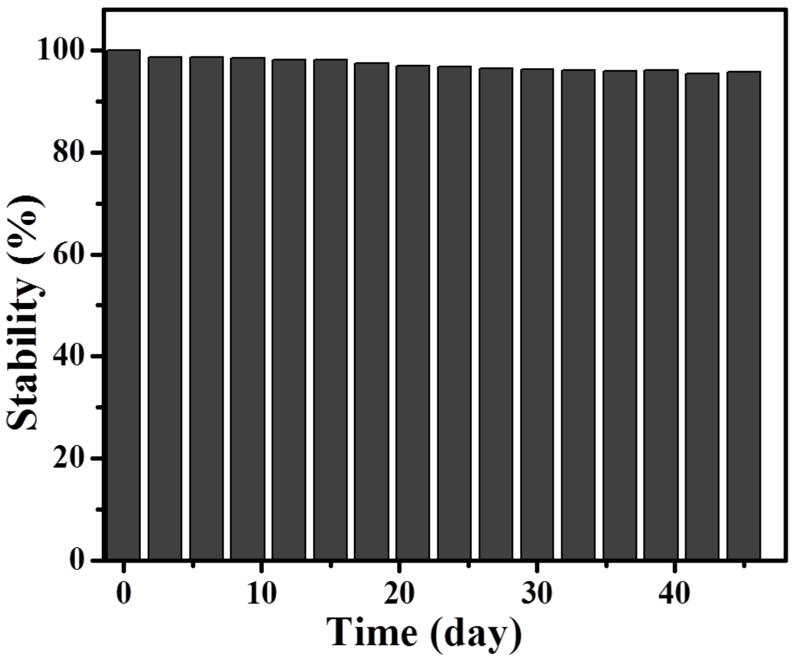

Supplementary Figure S6. Histogram showing stability test of biosensor electrode in 1.0 mM glucose for 45 days.

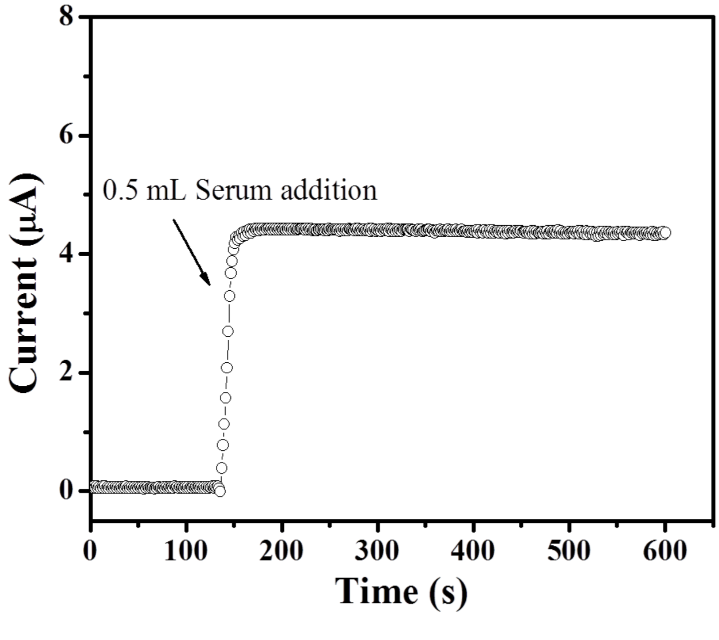

Supplementary Figure S7. Stability test of the fabricated electrode in serum sample.

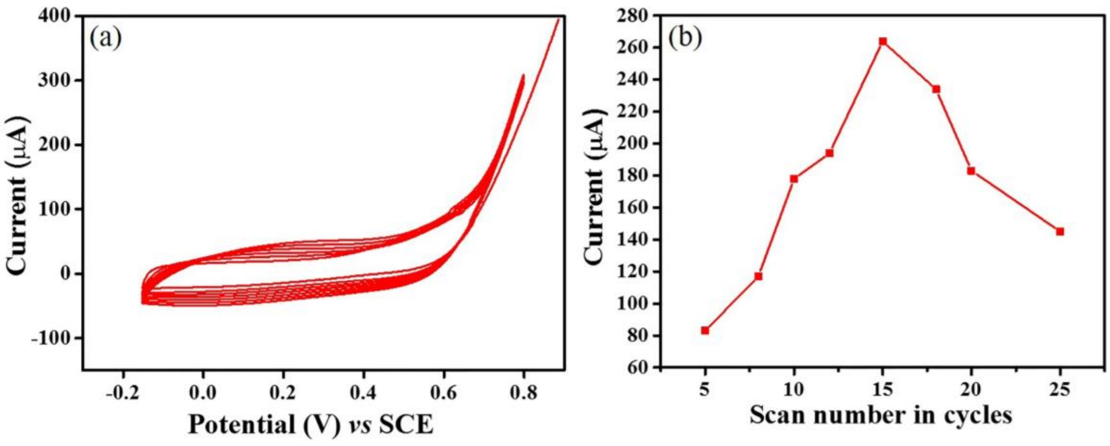

Supplementary Figure S8. (a) CVs obtained from Nf-GOx-*f*MWCNTs-PPy composite film on Pt disk electrode during *in situ* electrochemical polymerization. (b) Effect of scan numbers on anodic peak currents recorded from Nf-GOx-*f*MWCNTs-PPy/Pt biosensor in 0.1M PBS (pH 7.4) in the presence of 0.05 mM glucose.

References

1. Siswana, M. P., Ozoemena, K.I. & Nyokong T. Electrocatalysis of Asulam on Cobalt Phthalocyanine Modified Multi-Walled Carbon Nanotubes Immobilized on A Basal Plane Pyrolytic Graphite Electrode. Electrochemica. Acta **52,** 114-122 (2006).

2. Bard, A.J. & Faulkner, L.R. Electrochemical Methods: Fundamentals and Applications. Wiley & Sons 2000:

3. Konopka, S.J. & McDuffie B. Diffusion Coefficients of Ferri- and Ferrocyanide Ions in Aqueous Media, Using Twin-Electrode Thin-Layer Electrochemistry. Anal. Chem. **42,** 1741-1746 (1970).

4. Bai, L. et al. Direct Electrochemistry and Electrocatalysis of A Glucose Oxidase-Functionalized Bioconjugate as A Trace Label for Ultrasensitive Detection of Thrombin. Chem.Commun. **48,** 10972-10974 (2012).
